# A role for axon–glial interactions and Netrin-G1 signaling in the formation of low-threshold mechanoreceptor end organs

**DOI:** 10.1073/pnas.2210421119

**Published:** 2022-10-17

**Authors:** Shan Meltzer, Katelyn C. Boulanger, Emmanuella Osei-Asante, Annie Handler, Qiyu Zhang, Chie Sano, Shigeyoshi Itohara, David D. Ginty

**Affiliations:** ^a^Department of Neurobiology, Harvard Medical School, Boston, MA 02115;; ^b^HHMI, Harvard Medical School, Boston, MA 02115;; ^c^Laboratory for Behavioral Genetics, RIKEN Center for Brain Science, Wako, Saitama, 351-0198, Japan

**Keywords:** lanceolate complex, development, Netrin-G1, pruning, axon–glia interaction

## Abstract

Low-threshold mechanoreceptors (LTMRs) and their cutaneous end organs convert light mechanical forces acting on the skin into electrical signals that propagate to the central nervous system. In mouse hairy skin, hair follicle–associated longitudinal lanceolate complexes, which are end organs comprising LTMR axonal endings that intimately associate with terminal Schwann cell (TSC) processes, mediate LTMR responses to hair deflection and skin indentation. Here, we characterized developmental steps leading to the formation of Aβ rapidly adapting (RA)-LTMR and Aδ-LTMR lanceolate complexes. During early postnatal development, Aβ RA-LTMRs and Aδ-LTMRs extend and prune cutaneous axonal branches in close association with nascent TSC processes. Netrin-G1 is expressed in these developing Aβ RA-LTMR and Aδ-LTMR lanceolate endings, and *Ntng1* ablation experiments indicate that Netrin-G1 functions in sensory neurons to promote lanceolate ending elaboration around hair follicles. The Netrin-G ligand (NGL-1), encoded by *Lrrc4c*, is expressed in TSCs, and ablation of *Lrrc4c* partially phenocopied the lanceolate complex deficits observed in *Ntng1* mutants. Moreover, NGL-1–Netrin-G1 signaling is a general mediator of LTMR end organ formation across diverse tissue types demonstrated by the fact that Aβ RA-LTMR endings associated with Meissner corpuscles and Pacinian corpuscles are also compromised in the *Ntng1* and *Lrrc4c* mutant mice. Thus, axon–glia interactions, mediated in part by NGL-1–Netrin-G1 signaling, promote LTMR end organ formation.

## Significance

Our sense of touch is essential for fundamental tasks ranging from object recognition to social exchange. Yet, touch remains one of the least understood senses at the developmental level. Here, we investigate the formation of lanceolate complexes, which are mechanosensory end organs associated with hair follicles. The axons of touch sensory neurons innervating hairy skin extend into the skin at late embryonic and neonatal times, prune excessive branches during early postnatal development, and closely associate with nonmyelinating glial cells, called terminal Schwann cells (TSCs) during formation of lanceolate complexes. Moreover, NGL-1 and its receptor Netrin-G1 mediate a molecular dialogue between nascent TSCs and sensory neuron axonal endings to promote mechanosensory end organ formation in both hairy and nonhairy skin.

Touch sensation is an integral component of our sensory experience, allowing us to perceive and respond to the physical world. Light touch is mediated by morphologically and physiologically distinct classes of low-threshold mechanosensory neurons (LTMRs), which detect a range of innocuous tactile stimuli and convey their signals from the skin to the central nervous system (1, 2). The cell bodies of LTMRs are located in dorsal root ganglia (DRG) and cranial sensory ganglia. LTMRs have one axonal branch that extends to the skin and associates with different end organs and another branch that projects to the central nervous system and forms synapses onto second-order neurons in the spinal cord dorsal horn and brainstem (3). LTMRs have been classified as Aβ-, Aδ-, or C-LTMRs based on their action potential conduction velocities (4, 5). Aβ-LTMRs are heavily myelinated and Aδ-LTMRs are lightly myelinated, exhibiting rapid and intermediate conduction velocities, respectively. C-LTMRs are unmyelinated and have a slow conduction velocity. LTMRs are also classified as slowly, intermediately, or rapidly adapting (SA-, IA-, and RA-, respectively) according to their firing patterns in response to sustained indentation of the skin (3).

LTMR subtypes exhibit distinct intrinsic physiological properties and unique axonal endings associated with end organ structures across different skin types. The cutaneous axonal endings of Aβ RA-LTMRs form longitudinal lanceolate endings that enwrap hair follicles in hairy skin, terminate within Meissner corpuscles in glabrous (nonhairy) skin, or form Pacinian corpuscles located in the deep dermis or around bones (3). In mouse back hairy skin, Aβ RA-LTMR lanceolate endings form around guard hairs, which account for ∼1% of back skin hairs, and awl/auchene hairs. Aδ-LTMRs and C-LTMRs also form lanceolate endings, but unlike Aβ RA-LTMRs they are associated exclusively with nonguard hairs (awl/auchene and zigzag hairs) (6). These lanceolate ending structures are assumed to endow LTMRs with high sensitivity to hair deflection, skin indentation, and skin stroking (3). Investigating the formation of lanceolate endings associated with guard hairs and nonguard hairs will provide insights into mechanisms of development and regeneration of sensory neurons and their end organs that underlie touch. Somatosensory neuron axon terminals are encased by terminal Schwann cells (TSCs), which are a specialized group of nonmyelinating Schwann cells (2, 7). Indeed, light and electron microscopic studies reveal that lanceolate endings are arranged parallel to the hair follicle’s long axis, and each lanceolate ending is surrounded by TSC processes (8–11). Similar to lanceolate endings in hairy skin, the LTMR endings associated with Meissner corpuscles and Pacinian corpuscles are also wrapped by nonmyelinating Schwann cells, called lamellar cells (12–18).

LTMR innervation of hairy skin occurs in parallel with skin and hair follicle morphogenesis (19–22). Beginning on approximately embryonic day 14.5 (E14.5), primary guard hair keratinocyte precursor cells elongate to form hair follicle placodes and then proliferate and invaginate to form hair follicles (23). Secondary hair follicle development occurs in two waves: awl/auchene hairs develop at approximately E16.5, and zigzag hairs develop around birth (E18–postnatal day 1 [P1]) (24). Developing hair follicles can release extrinsic cues to instruct the formation of lanceolate complexes. For example, keratinocytes on the caudal side of hair follicles express BDNF and control the polarized targeting of TrkB-expressing Aδ-LTMR endings to the caudal side of hair follicles (25). Moreover, hair follicle epidermal stem cells deposit EGFL6, an ECM protein, into the collar matrix, to regulate the proper patterning of lanceolate complexes (26). However, the precise timing of LTMR innervation of hair follicles, whether LTMR axons undergo pruning during hair follicle innervation, the nature of the relationship between developing lanceolate endings and nascent TSCs, and molecular cues that instruct lanceolate ending morphological maturation remain unexplored.

Netrin-G1, encoded by *Ntng1*, is a member of the family of glycosyl-phosphatidylinositol (GPI)-anchored cell adhesion molecules. Netrin-G1 can promote synapse formation, microglial accumulation along axons, axonal outgrowth, and laminar organization of dendrites (27–30). Moreover, Netrin-G1 and its relative Netrin-G2 can localize to presynaptic membranes and instruct the specificity in synaptic connectivity (28, 30–32). In these contexts, Netrin-G1 is considered to function as a cell surface receptor. The Netrin-G1 ligand, NGL-1, which is encoded by *Lrrc4c*, belongs to a family of postsynaptic adhesion molecules (27, 29, 32–34). Mutations in both *Ntng1* and *Lrrc4c* have been implicated in neurological diseases, including Rett syndrome, schizophrenia, and autism (35–38). Yet, the functions of Netrin-G1 and NGL-1 in peripheral nervous system development have not been established.

Here, we used mouse genetic approaches to visualize Aβ RA-LTMRs and Aδ-LTMRs during late embryonic and early postnatal development (25, 39), which allowed us to define the timing of hairy skin innervation and formation of their lanceolate complexes. Aβ RA-LTMR and Aδ-LTMR peripheral innervation patterns are established late embryonically and neonatally, exuberant axonal branches are pruned around birth, and newly formed lanceolate endings associate intimately with nascent TSCs. We found that Netrin-G1 signaling functions in somatosensory neurons to promote proper formation of Aβ RA-LTMR and Aδ-LTMR lanceolate complexes. *Lrrc4c*, encoding the Netrin-G1 ligand, NGL-1, is expressed in developing TSCs in hairy skin, and its deletion leads to similar, albeit milder, Aβ RA-LTMR and Aδ-LTMR lanceolate ending deficits. Moreover, we observed aberrant Meissner corpuscle and Pacinian corpuscle development in the absence of NGL-1–Netrin-G1 signaling. Our findings delineate LTMR end organ developmental stages and reveal a role for NGL-1–Netrin-G1 signaling between TSCs and LTMR endings in mechanoreceptor end organ formation.

## Results

### Hair Follicle Innervation and Pruning of Aβ RA-LTMR and Aδ-LTMR Axons in Hairy Skin.

**Fig. 1.**
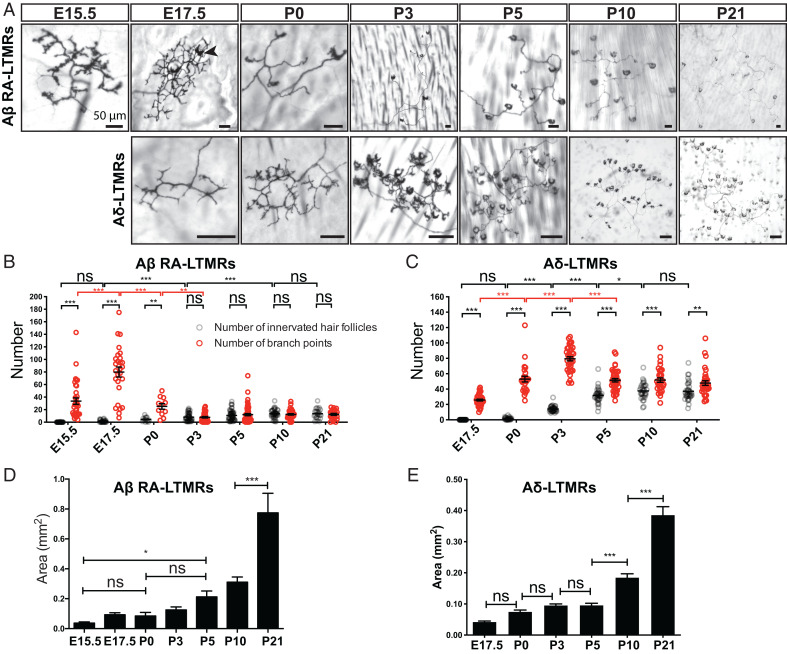
Peripheral arbors of Aβ RA-LTMRs and Aδ-LTMRs undergo pruning around birth. (*A*) Wholemount alkaline phosphatase staining of back hairy skin to visualize peripheral terminals of individual Aβ RA-LTMRs and Aδ-LTMRs at different developmental time points and at P21. The black arrowhead points to one example of a crescent-shaped axonal ending wrapped around a hair follicle. (Scale bars, 50 μm.) (*B* and *C*) Quantification of the number of innervated hair follicles (gray) and branch points (red) of individual Aβ RA-LTMRs (*B*, *n* = 257 neurons from 37 animals) and Aδ-LTMRs (*C*, *n* = 210 neurons from 17 animals). Each dot represents a single neuron. Two-way ANOVA with Tukey's multiple comparisons test and Šídák's multiple comparisons test. (*D* and *E*) Quantification of the area of skin covered by individual Aβ RA-LTMRs (*D*) and Aδ-LTMRs (*E*). One-way analysis of variance (ANOVA) with Šídák's multiple comparisons test. ns, not significant, *P < 0.05, **P < 0.01, ***P < 0.001.

We first investigated the timing of Aβ RA-LTMR and Aδ-LTMR innervation of hairy skin. For this, Aβ RA-LTMRs and Aδ-LTMRs were sparsely labeled by crossing Cre-dependent *Brn3a^f(AP)^* alkaline phosphatase reporter mice (40) with *Ret^CreER^* or *TrkB^CreER^* mice, respectively (25, 39). To label developing Aβ RA-LTMR and Aδ-LTMR endings, pregnant females were treated with tamoxifen at E11.5 and E12.5, respectively. At E15.5, 1 d after guard hairs emerge, Aβ RA-LTMR axons were observed in the skin where they exhibited many small protrusions extending from the major branches, but they had not yet innervated nascent hair follicles (Fig. 1*A*). Aβ RA-LTMR innervation of hair follicles was observed beginning ∼E17.5; these endings appeared as crescent-shaped axonal wrappings around nascent hair follicles. Many Aβ RA-LTMR axonal branches were observed at E17.5; most of these were pruned by P3, a time point at which the number of branch points did not significantly differ from the number of innervated hair follicles (Fig. 1*B*). In contrast, relative to Aβ RA-LTMRs, Aδ-LTMRs exhibited delayed innervation of hair follicles, as no Aδ-LTMR follicle innervation was observed until ∼P0 (Fig. 1 *A* and *C*). As with Aβ RA-LTMRs, Aδ-LTMRs extended extranumerary branches during their period of active hair follicle innervation at ∼P3, and branches that did not associate with hair follicles were pruned by P5 (Fig. 1*C*). After P5, the number of branch points was still significantly more than the number of innervated hair follicles (Fig. 1*C*), presumably because some hair follicles were innervated by more than one axonal branch of the same Aδ-LTMR neuron (*SI Appendix*, Fig. S1*A*) (41). Both Aβ RA-LTMRs and Aδ-LTMRs gradually increased the size of their innervation area during postnatal development as skin expansion occurred during growth of the pups (Fig. 1 *D* and *E*).

### Nascent Aβ RA-LTMR and Aδ-LTMR Lanceolate Endings Closely Interact with Terminal Schwann Cells during Hair Follicle Innervation.

**Fig. 2.**
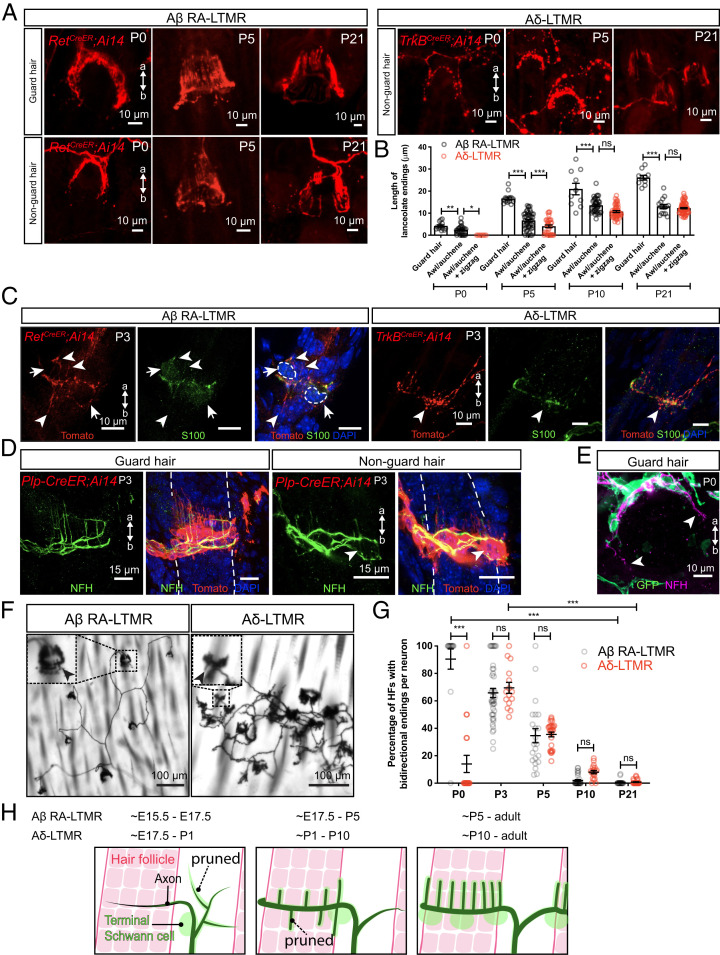
Lanceolate endings of Aβ RA-LTMRs and Aδ-LTMRs emerge around hair follicles neonatally and closely associate with terminal Schwann cells. (*A*) Wholemount immunostaining images of back hairy skin showing Aβ RA-LTMRs in *Ret^CreER^; Ai14* animals and Aδ-LTMRs in *TrkB^CreER^; Ai14* animals at P0, P5, and P21. Guard hairs were identified by TROMA-I (Krt8) staining that labels Merkel cells that assemble into touch domes. (*B*) Quantification of the length of lanceolate endings associated with hair follicles. Aβ RA-LTMRs (black) and Aδ-LTMRs (red) at P0, P5, P10, and P21, showing that Aβ RA-LTMR lanceolate endings innervating guard hairs develop earlier than the lanceolate endings innervating awl/auchene and zigzag hairs (together grouped as nonguard hairs). Aβ RA-LTMRs (*n* = 3 animals per time point): P0 (14 guard hairs and 28 nonguard hairs), P5 (14 guard hairs and 47 nonguard hairs), P10 (10 guard hairs and 39 nonguard hairs), and P21 (10 guard hairs and 15 nonguard hairs). Aδ-LTMRs (*n* = 3 animals per time point): P0 (8 nonguard hairs), P5 (32 nonguard hairs), P10 (49 nonguard hairs), and P21 (68 nonguard hairs). Each dot represents lanceolate ending length around a hair follicle. One-way ANOVA was used for each time point. (*C*) Back hairy skin sections from *Ret^CreER^; Ai14* animals (*n* = 3 animals) and *TrkB^CreER^; Ai14* animals (*n* = 3 animals) were stained with an anti-Tomato antibody to label Aβ RA-LTMRs and Aδ-LTMR axonal terminals around hair follicles at P3. S100 (green) staining labels TSCs, and DAPI labels nuclei. White arrowheads point to some of the nascent lanceolate endings growing toward the apical and basal sides of the skin. White arrows and dotted lines denote cell bodies of TSCs. (*D*) Wholemount immunostaining images from *Plp-CreER; Ai14* animals (back hairy skin, *n* = 3 animals), which were stained with an anti-Tomato antibody to label TSCs at P3. NFH (green) labels lanceolate endings of Aβ RA-LTMRs. White arrowhead points to nascent lanceolate endings growing toward the basal side of the skin. (*E*) Wholemount immunostaining image from a *PLP-EGFP* animal (back hairy skin, *n* = 3 animals), which was stained with an anti-GFP antibody to label TSCs at P0. White arrowhead points to the growing ends of the circumferential endings around the guard hair. (*F*) Wholemount alkaline phosphatase staining of back hairy skin reveals peripheral terminals from individual Aβ RA-LTMRs and Aδ-LTMRs sparsely labeled at P3. *Insets* showing a high-magnification region of lanceolate endings around hair follicles. Black arrowheads point to nascent lanceolate endings extending toward the basal side of the skin. (*G*) Quantification of the percentage of hair follicles with bidirectional endings per neuron for Aβ RA-LTMRs and Aδ-LTMRs at P0, P5, P10, and P21. Aβ RA-LTMRs: P0 (14 neurons from three animals), P3 (42 neurons from four animals), P5 (22 neurons from six animals), P10 (32 neurons from three animals), and P21 (21 neurons from three animals). Aδ-LTMRs: P0 (19 neurons from three animals), P3 (15 neurons from three animals), P5 (27 neurons from four animals), P10 (19 neurons from three animals), and P21 (27 neurons from three animals). HF, hair follicle. Each dot represents the percentage of hair follicles with bidirectional endings measured for a single neuron. Two-way ANOVA with Tukey's multiple comparisons test and Šídák's multiple comparisons test. (*H*) Summary of peripheral innervation steps for Aβ RA-LTMRs and Aδ-LTMRs. Black represents sensory axons, green cells represent terminal Schwann cells, and hair follicles are depicted in red. Dotted lines show that some sensory neuron branches and lanceolate endings are pruned during development. a, apical; b, basal. ns, not significant, *P < 0.05, **P < 0.01, ***P < 0.001.

We next examined the timing of Aβ RA-LTMR and Aδ-LTMR lanceolate complex formation. To visualize lanceolate endings around guard and nonguard hairs, we combined *Rosa26^LSL-tdTomato^* (Ai14) with *Ret^CreER^* and *TrkB^CreER^* mice (25, 39) and performed wholemount immunostaining at different time points during postnatal development (Fig. 2*A*). At P0, nascent Aβ RA-LTMR lanceolate endings were observed around guard and nonguard hair follicles, while Aδ-LTMRs only formed crescent endings around nonguard hair follicles and did not yet exhibit lanceolate endings (Fig. 2 *A* and *B*). Lanceolate endings around guard hairs were already significantly longer than those around nonguard hairs, which is consistent with the earlier formation and maturation of guard hairs (Fig. 2*B*). By P5, lanceolate endings from both Aβ RA-LTMRs and Aδ-LTMRs were observed, and lanceolate endings around guard hairs were significantly longer than those around nonguard hairs (Fig. 2*B*). By P10, the length of Aβ RA-LTMR lanceolate endings around nonguard hairs was comparable to those of Aδ-LTMRs. Lanceolate endings continued to extend during postnatal development and achieved mature morphology by P21 (Fig. 2 *A* and *B*).

Mature LTMR lanceolate endings are closely associated with TSCs, whose cell bodies reside at the base of lanceolate complexes (11, 42). To visualize the temporal and morphological relationships between lanceolate axonal endings and TSCs during lanceolate complex formation, wholemount immunostaining with S100, a peripheral glia marker, and tdTomato was performed using skin from P3 *Ret^CreER^; Rosa26^LSL-tdTomato^* and *TrkB^CreER^; Rosa26^LSL-tdTomato^* animals. Although S100 immunolabeling was faint at P3, some Aβ RA-LTMR and Aδ-LTMR lanceolate endings were closely associated with this glial marker at this time point (Fig. 2*C*). As a complementary approach, *Plp-CreER; Rosa26^LSL-tdTomato^* animals were generated to genetically label TSCs and visualize their processes. In the presence of tamoxifen, *Plp-CreER*, driven by the mouse *Plp1*, proteolipid protein (myelin) 1 promoter, activates the expression of tdTomato in Schwann cells (43). Similar to lanceolate axons, TSCs exhibited exuberant processes around hair follicles at P3. Neurofilament H (NFH)-positive Aβ RA-LTMR lanceolate endings associated with both guard and nonguard hairs were closely associated with TSC processes at this age (Fig. 2*D*), with 97.9% of NFH^+^ lanceolate endings around guard hairs enwrapped by TSC protrusions (140 lanceolate endings from three animals). To examine the axon–glial interaction at an earlier time point, we examined the back hairy skin in P0 *PLP-EGFP* animals (44) when Aβ RA-LTMR axons are actively wrapping guard hair follicles with circumferentially oriented axons and extending nascent lanceolate endings (Fig. 2*A*). Interestingly, while the growing ends of the circumferential NFH^+^ axons are not associated with GFP^+^ TSCs (observed in 18 out of 19 guard hairs from three animals) (Fig. 2*E*), 85.8% of the nascent lanceolate endings are associated with TSCs (135 lanceolate endings from three animals) at this time point. This result suggests that circumferentially oriented axons of the lanceolate complex may lead TSCs to their target locations around hair follicles to support lanceolate ending formation.

### Aβ RA-LTMRs and Aδ-LTMRs Prune Basal-Orienting Lanceolate Endings during Early Postnatal Development.

Mature lanceolate endings orient exclusively toward the apical side of the epidermis (Fig. 2*A*) (11, 25, 42). Interestingly, we frequently observed lanceolate endings extending toward the basal side of the epidermis in P3 skin (Fig. 2 *C* and *D*), suggesting that these basal-orienting lanceolate endings are pruned later during postnatal development. To assess the prevalence and elimination of these basal-orienting lanceolate endings, we examined sparsely labeled Aβ RA-LTMRs and Aδ-LTMRs in hairy skin using wholemount alkaline phosphatase labeling and high-magnification visualization. We quantified the percentage of hair follicles with lanceolate endings pointing to both apical and basal sides of the epidermis (bidirectional lanceolate endings) for individual neurons across postnatal development (Fig. 2 *F* and *G*). At P21, lanceolate endings wrapping around hair follicles pointed exclusively toward the apical side of the epidermis (Fig. 1*A*). Conversely, at P0, most hair follicles innervated by Aβ RA-LTMRs exhibited lanceolate endings with both apical and basal orientations. Moreover, although at P0 most Aδ-LTMRs had just begun to extend lanceolate endings, those that did often displayed bidirectional orientation (Fig. 2*G*). The percentages of hair follicles with bidirectional endings gradually decreased for both Aβ RA-LTMRs and Aδ-LTMRs during subsequent postnatal development and bidirectional endings were eventually gone by P21 (Fig. 2*G*). The temporal similarity in the pruning of basal-orienting endings across these two lanceolate ending neurons suggests that there may be a common cue regulating the elimination of basal-orienting lanceolate endings.

Thus, despite a difference in their timing of skin innervation, Aβ RA-LTMRs and Aδ-LTMRs have similar developmental features (Fig. 2*H*). First, both populations exhibit excessive branching and prune axonal branches that do not innervate hair follicles, with Aβ RA-LTMRs undergoing pruning earlier than Aδ-LTMRs. Second, both LTMRs actively extend axons to innervate hair follicles while greatly expanding their peripheral arbor area during growth of the body. Third, both have nascent lanceolate protrusions that associate intimately with TSC processes. Fourth, both exhibit apical- and basal-oriented lanceolate extensions and eliminate the basal extensions to achieve their mature morphological properties by P21.

### Netrin-G1 Is Expressed in Developing and Mature Aβ RA-LTMRs and Aδ-LTMRs.

To begin to test the hypothesis that common molecular mechanisms may function across LTMR types during the assembly of lanceolate complexes, we searched published datasets for cell-adhesion molecules and axon guidance proteins expressed in developing Aβ RA-LTMRs and Aδ-LTMRs, but not in nociceptors or proprioceptors, which do not form lanceolate complexes (45, 46). Netrin-G1 stood out in this analysis because it is expressed in both Aβ RA-LTMRs and Aδ-LTMRs throughout development and in adulthood (45, 46). Netrin-G1 plays critical roles in many aspects of neural development, including functioning as a synaptic cell adhesion molecule to promote excitatory synapse formation, regulating formation of dendritic laminar structures, and axonal outgrowth (27, 28, 30, 32). Thus, we hypothesized that Netrin-G1 may orchestrate LTMR lanceolate ending complex formation or other features of Aβ RA-LTMR and Aδ-LTMR axonal development.

**Fig. 3.**
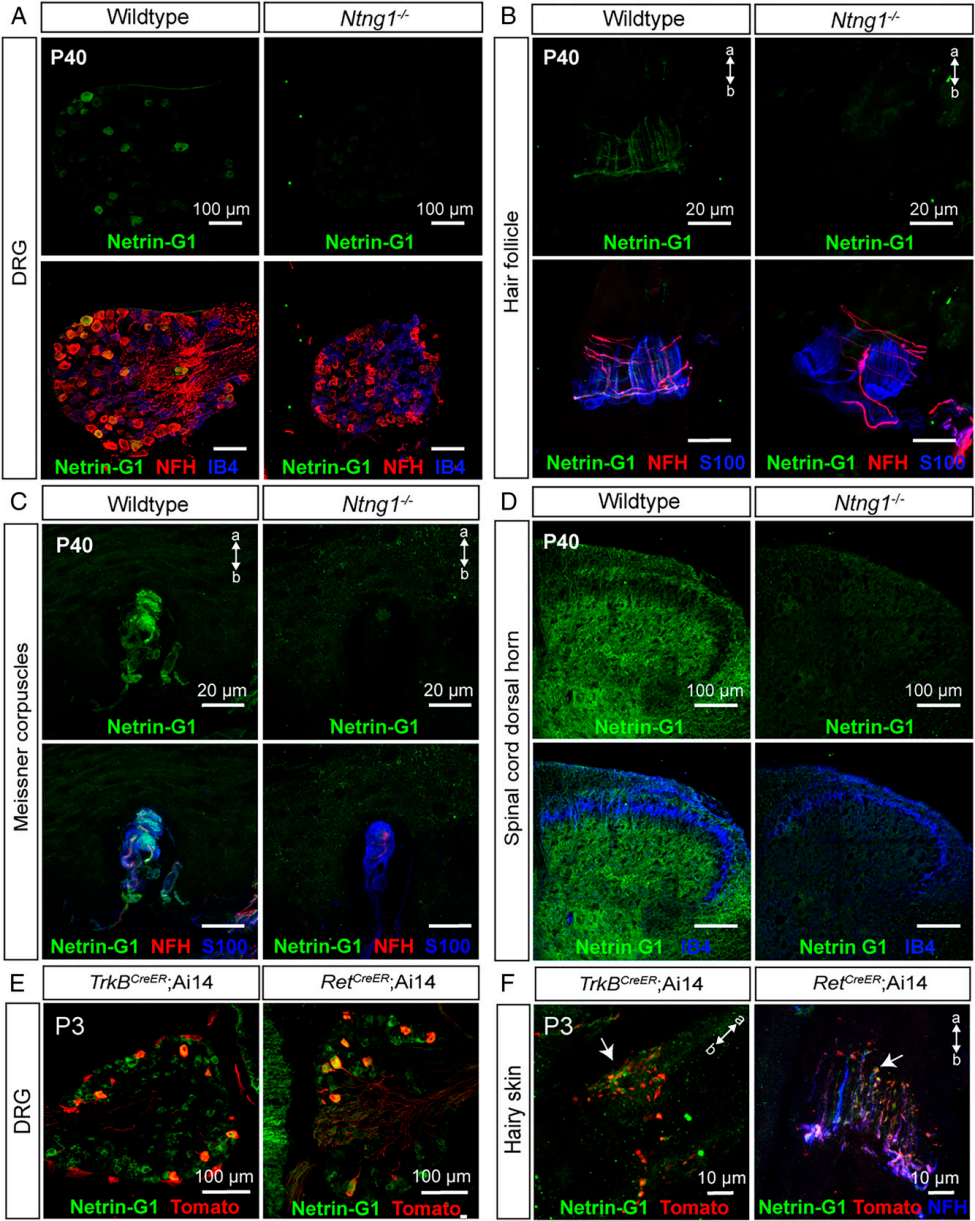
Netrin-G1 is expressed in developing and adult Aβ RA-LTMRs and Aδ-LTMRs. (*A*–*D*) Immunohistochemistry (IHC) images of T11–T13 DRG sections from wild-type and *Ntng1^−/−^* animals (*n* = 3 animals per genotype) at P40, showing expression of Netrin-G1 in NFH^+^ DRG neurons (*A*), lanceolate and circumferential endings around hair follicles (*B*), Aβ RA-LTMRs innervating Meissner corpuscles (*C*), and lumbar (L4) spinal cord (*D*). Netrin-G1 signal (green) is absent in *Ntng1^−/−^* animals. IB4 labels a large subset of nonpeptidergic sensory neurons and S100 labels TSCs. (*E* and *F*) Thoracic DRG and back hairy skin sections obtained from P3 *Ret^CreER^; Ai14* animals (*n* = 3 animals) and *TrkB^CreER^; Ai14* animals (*n* = 3 animals) were stained with Netrin-G1 and Tomato to visualize Aβ RA-LTMRs and Aδ-LTMRs. NFH (blue) labels lanceolate endings of Aβ RA-LTMRs. a, apical; b, basal.

We first verified expression of Netrin-G1 in P40 LTMRs using a Netrin-G1 antibody (47). All Netrin-G1^+^ DRG cells were large diameter and NFH^+^, consistent with their expression in NFH^+^ Aβ RA-LTMRs and Aδ-LTMRs (Fig. 3*A*). As a control for Netrin-G1 antibody specificity, we used *Ntng1^−/−^* mutants and observed no antibody signal (Fig. 3 *A*–*D*). Netrin-G1 protein was also detected in lanceolate endings around hair follicles as well as Aβ RA-LTMR axon terminals in Meissner corpuscles of glabrous skin (Fig. 3 *B* and *C*). Netrin-G1 signal was also detected in Aβ field-LTMR circumferential endings in hairy skin and at a low level in lamellar cells associated with Meissner corpuscles (Fig. 3 *B* and *C*). In addition, Netrin-G1 was detected in the spinal cord, with the highest signal in lamina III and IV, and the lowest signal in lamina II (marked by IB4). The signal observed below lamina II is consistent with the central targeting region of Aβ RA-LTMRs and Aδ-LTMRs (Fig. 3*D*). To ask whether Netrin-G1 is expressed in developing Aβ RA-LTMRs and Aδ-LTMRs, we performed Netrin-G1 immunostaining with DRGs and skin from *Ret^CreER^; Ai14* and *TrkB^CreER^; Ai14* animals. Indeed, Netrin-G1 was present in both the cell bodies and lanceolate endings of genetically labeled Aβ RA-LTMRs and Aδ-LTMRs at P3 (Fig. 3 *E* and *F*). Thus, Netrin-G1 is expressed in developing Aβ RA-LTMRs and Aδ-LTMRs and localizes to their axonal endings during the period of lanceolate ending maturation.

### Netrin-G1 Regulates Lanceolate Ending Formation in Aβ RA-LTMRs and Aδ-LTMRs.

**Fig. 4.**
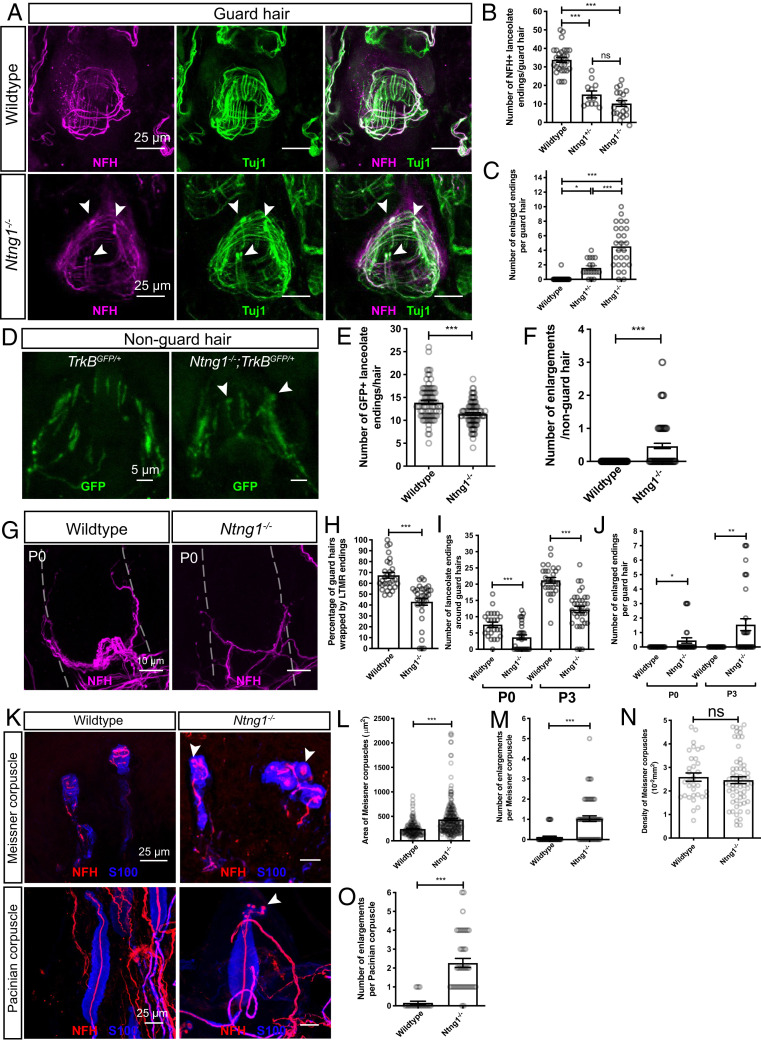
Netrin-G1 regulates the formation of lanceolate endings, Meissner corpuscles, and Pacinian corpuscles. (*A*) Wholemount immunostaining images of guard hairs in back hairy skin of adult wild-type and *Ntng1^−/−^* animals. Aβ RA-LTMRs lanceolate endings are marked by NFH (magenta) and Tuj1 (green) labeling. (*B* and *C*) Quantification of the number of NFH^+^ lanceolate endings (*B*) and the number of enlarged endings (*C*) per guard hair for Aβ RA-LTMRs in wild-type (*B*: 29 hair follicles from five animals; *C*: 42 hair follicles from seven animals), *Ntng1^+/−^* (*B*: 12 hair follicles from two animals; *C*: 17 hair follicles from two animals) and *Ntng1^−/−^* (*B*: 19 hair follicles from three animals; *C*: 26 hair follicles from three animals) animals, showing fewer Aβ RA-LTMR lanceolate endings in *Ntng1^+/−^* and *Ntng1^−/−^* animals. Each dot represents a single hair follicle. One-way ANOVA test. (*D*) Wholemount immunostaining images of nonguard hairs in back hairy skin in *TrkB^GFP/+^* and *Ntng1^−/−^; TrkB^GFP/+^* animals. Aδ-LTMRs lanceolate endings are marked by GFP labeling. (*E* and *F*) Quantification of the number of GFP^+^ lanceolate endings (*E*) and the number of enlarged endings (*F*) in *TrkB^GFP/+^* (90 hair follicles from three animals) and *Ntng1^−/−^; TrkB^GFP/+^* (95 hair follicles from three animals) animals, showing similar deficits in lanceolate ending formation with nonguard hairs. Student’s unpaired *t* test. (*G*) Wholemount immunostaining images of guard hairs in back hairy skin of P0 wild-type and *Ntng1^−/−^* animals. (*H*) Quantification of the percentages of guard hairs wrapped by NFH^+^ lanceolate endings at P0 in wild-type (29 hair follicles from three animals) and *Ntng1^−/−^* (33 hair follicles from three animals) animals. Student’s unpaired *t* test. (*I* and *J*) Quantification of the number of NFH^+^ lanceolate endings (*I*) and the number of enlarged endings (*J*) per guard hair in back hairy skin for Aβ RA-LTMRs in wild-type (25 hair follicles from three animals for P0; 28 hair follicles from five animals for P3) and *Ntng1^−/−^* (31 hair follicles from three animals for P0; 25 hair follicles from five animals for P3) animals. Student’s unpaired *t* test. (*K*) Representative IHC images of forepaw glabrous skin sections and Pacinian corpuscles. Meissner corpuscles and Pacinian corpuscles are labeled by S100 (blue) for visualizing lamellar cells and NFH (red) for visualizing Aβ RA-LTMRs. Arrowheads point to axonal enlargements. (*L*–*N*) Quantification of the area (*L*), number of enlargements (*M*), and density (*N*) of Meissner corpuscles in the epidermis of wild-type (44 skin sections from four animals) and *Ntng1^−/−^* (60 skin sections from four animals) mice. Each dot represents a single skin section (*O*). Student’s unpaired *t* test. (*O*) Quantification of the number of enlargements per Pacinian corpuscle from wild-type (19 corpuscles from three animals) and *Ntng1^−/−^* (41 corpuscles from three animals) mice. Student’s unpaired *t* test. Each dot represents a single Pacinian corpuscle. ns, not significant, *P < 0.05, **P < 0.01, ***P < 0.001.

We next tested the hypothesis that Netrin-G1 contributes to Aβ RA-LTMR skin innervation and lanceolate complex formation using *Ntng1* knockout mice. For this, lanceolate endings around guard hairs of P40 *Ntng1^−/−^*, *Ntng1^+/−^*, and littermate control mice were visualized with neurofilament heavy chain (NFH) and tubulin β-3 (Tuj1; all sensory axons) antibody immunostaining (Fig. 4*A* and *SI Appendix*, Fig. S1 *B* and *C*). The number of lanceolate endings around guard hairs in *Ntng1^−/−^* mice was reduced compared to controls (Fig. 4*B* and *SI Appendix*, Fig. S1*C*). A comparable number of NFH^+^ neurons was observed in DRGs of *Ntng1^−/−^* and control mice, suggesting that the reduction of lanceolate endings was not caused by loss of DRG neurons (*SI Appendix*, Fig. S2). Moreover, lanceolate endings in *Ntng1^−/−^* mice showed a substantial number of enlargements in their distal tip regions (Fig. 4 *A* and *C*). In addition, *Ntng1^+/−^* mice exhibited an intermediate phenotype in both the number of lanceolate endings and the number of enlarged endings around guard hairs, suggesting that a precise level of *Ntng1* expression is critical for lanceolate ending formation (Fig. 4 *B* and *C* and *SI Appendix*, Fig. S1). To determine whether similar deficits exist in Aδ-LTMRs, we visualized Aδ-LTMR lanceolate endings using *TrkB^GFP^* mice (25). As in Aβ RA-LTMRs, the number of Aδ-LTMR lanceolate endings was decreased and the number of lanceolate ending enlargements around nonguard hairs was increased in *Ntng1^−/−^* mice, indicating that Netrin-G1 regulates lanceolate complex formation in both Aβ RA-LTMRs and Aδ-LTMRs (Fig. 4 *D*–*F*).

To determine whether the paucity of lanceolate complexes in *Ntng1^−/−^* adult mice reflects a developmental alteration in lanceolate ending formation or a loss of mature lanceolate endings in adults, we next assessed the timing of hair follicle innervation deficits during postnatal development in the mutants. At P0, *Ntng1^−/−^* mice exhibited a significant reduction in the percentage of guard hair follicles wrapped by Aβ RA-LTMRs (Fig. 4 *G* and *H*), in contrast to complete wrapping of guard hair follicles by circumferential axons of Aβ RA-LTMRs in adults (*n* = 27 guard hairs in three wild-type mice and *n* = 21 guard hairs in three *Ntng1^−/−^* mice). While control animals start to exhibit nascent lanceolate endings around guard hairs at P0 and continue at P3, lanceolate ending extension in *Ntng1^−/−^* mutants was significantly reduced at these times (Fig. 4*I*). Similarly, enlarged endings are present at both P0 and P3 (Fig. 4*J*). These findings indicate that Netrin-G1 controls lanceolate complex formation during neonatal development.

We also tested whether the reduced number of lanceolate endings in *Ntng1^−/−^* mice results from fewer axonal branches per neuron, a deficit in lanceolate complex formation in individual branches, or both. To address this, sparse neuronal labeling in *Ntng1^−/−^* mice was performed to visualize the morphological features of individual Aβ RA-LTMRs and Aδ-LTMR neurons in hairy skin at P3 (*SI Appendix*, Fig. S3*A*). Interestingly, *Ntng1^−/−^* mice exhibited a reduction in the number of innervated hair follicles per neuron (*SI Appendix*, Fig. S3*B*), consistent with the delay of guard hair wrapping by NFH^+^ endings observed at P0–P1 (Fig. 4*H*). However, the number of branch points and area of individual neurons in *Ntng1^−/−^* mice did not differ from controls (*SI Appendix*, Fig. S3 *C* and *D*). Thus, while dispensable for initial stages of Aβ RA-LTMR and Aδ-LTMR skin innervation and axonal branching patterns, Netrin-G1 controls LTMR axonal wrapping around hair follicle and lanceolate complex formation during neonatal and early postnatal development.

The hairy skin lanceolate ending deficits in *Ntng1^−/−^* mice prompted us to ask whether other LTMR end organs exhibit morphogenesis deficits in these mice (2, 3). Interestingly, Meissner corpuscles in *Ntng1^−/−^* mice appeared larger and their NFH^+^ axons displayed more enlargements than littermate controls (Fig. 4 *K*–*M*), although the density of Meissner corpuscles was not altered in the mutants (Fig. 4*N*). In wild-type Pacinian corpuscles, NFH^+^ Aβ RA-LTMR axons usually have one bulbous ultraterminal region (3, 16). However, Pacinian-innervating Aβ RA-LTMR axons in *Ntng1^−/−^* mice exhibited an increase in the number of enlargements in their ultraterminal region (Fig. 4 *K* and *O*). These axonal enlargements resemble those observed in the hair follicle lanceolate endings, suggesting a common role for Netrin-G1 signaling in the morphogenesis of Aβ RA-LTMR axons and their end organs.

### Netrin-G1 Functions in Somatosensory Neurons to Regulate the Formation of Lanceolate Endings and Aβ RA-LTMR–Innervating Corpuscles.

**Fig. 5.**
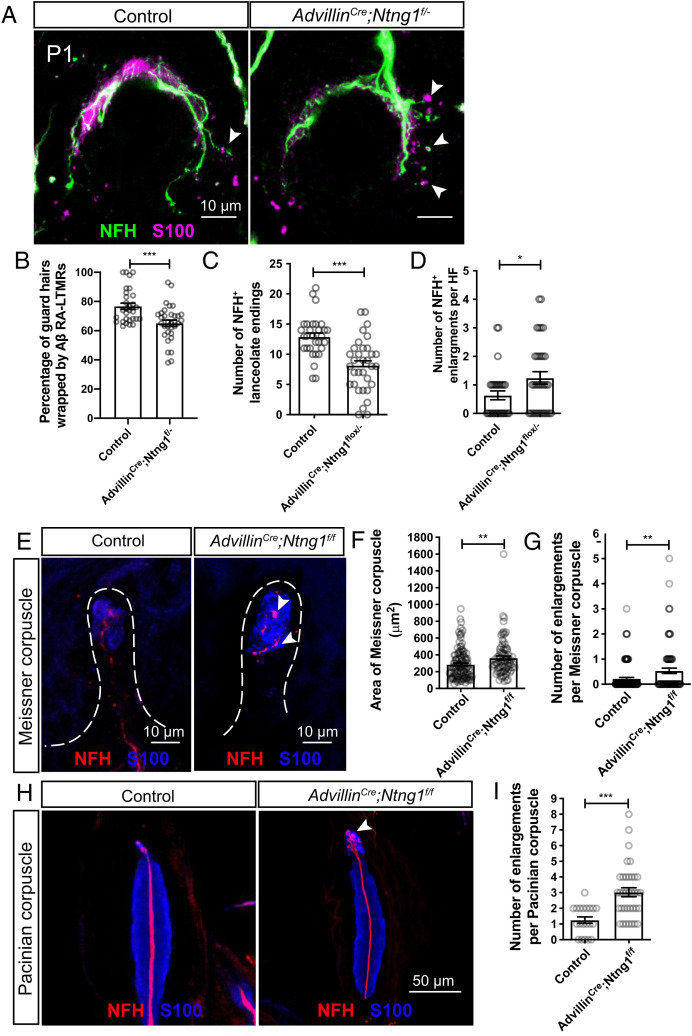
Netrin-G1 functions in somatosensory neurons to regulate mechanosensory end organ formation. (*A*) Wholemount IHC images of guard hairs in back hairy skin of P1 control (*Ntng1^+/−^*) and *Advillin^Cr^*^e^*; Ntng1^f/−^* animals. (*B*) Quantification of the percentage of guard hairs wrapped by NFH^+^ lanceolate endings of P1 control (30 hair follicles from three animals) and *Advillin^Cre^; Ntng1^f/−^* (33 hair follicles from three animals) animals. (*C* and *D*) Quantification of the number of NFH^+^ lanceolate endings (*C*) and the number of enlarged endings (*D*) per guard hair in P1 control (30 hair follicles from three animals) and *Advillin^Cre^; Ntng1^f/−^* (33 hair follicles from three animals) mice. (*E*) Representative IHC images of forepaw glabrous skin sections, showing Meissner corpuscles in control and *Advillin^Cre^; Ntng1^f/f^* mice. Arrowheads point to axonal enlargements. (*F* and *G*) Quantification of the area (*F*) and number of enlargements (*G*) of Meissner corpuscles in the epidermis of control (32 sections from three animals) and *Advillin^Cre^; Ntng1^f/f^* (31 sections from three animals) mice. (*H* and *I*) Representative IHC images of Pacinian corpuscles (*H*) and quantification of the number of enlargements per Pacinian corpuscle (*I*) in littermate control (20 corpuscles from two animals) and *Advillin^Cre^; Ntng1^f/f^* (36 corpuscles from three animals) mice. The arrowhead points to axonal enlargements. Each dot represents a single Pacinian corpuscle. Student’s unpaired *t* test. *P < 0.05, **P < 0.01, ***P < 0.001.

To determine whether Netrin-G1 functions in somatosensory neurons to promote lanceolate ending formation, Netrin-G1 was conditionally deleted from embryonic somatosensory neurons using *Advillin^Cre^* mice (48) and a conditional “floxed” *Ntng1* allele (49). To increase the efficiency of embryonic *Ntng1* deletion, we generated *Advillin^Cre^; Ntng1^f/−^* mutants and compared them to littermate controls carrying one copy of the null allele. As with *Ntng1^−/−^* mice (Fig. 4*I*), at P1, *Advillin^Cre^; Ntng1^f/−^* conditional mutants showed a delay in wrapping of lanceolate endings around guard hairs (Fig. 5 *A* and *B*). Similarly, the conditional mutants displayed fewer NFH^+^ lanceolate endings and many enlarged endings associated with lanceolate complexes around guard hairs (Fig. 5 *C* and *D*). These findings suggest that Netrin-G1 acts in somatosensory neurons to promote lanceolate complex formation. Moreover, as in *Ntng1^−/−^* mice, *Advillin^Cre^; Ntng1^f/f^* mice also exhibited an increase in the area of Meissner corpuscles and the number of axonal enlargements (Fig. 5 *E*–*G*) as well as an increase in the number of enlargements in axons in Pacinian corpuscles (Fig. 5 *H* and *I*). The phenotypes in the conditional knockouts are less severe than the germline null mutants, and this could reflect incomplete recombination in sensory neurons or a noncell autonomous function of Netrin-G1. Nevertheless, these findings indicate that Netrin-G1 acts in Aβ RA-LTMRs during formation of lanceolate endings associated with hair follicles, Meissner corpuscles of glabrous skin, and Pacinian corpuscles associated with the periosteum of bones.

### *Lrrc4c* Is Expressed in Myelinating Schwann Cells and Terminal Schwann Cells.

**Fig. 6.**
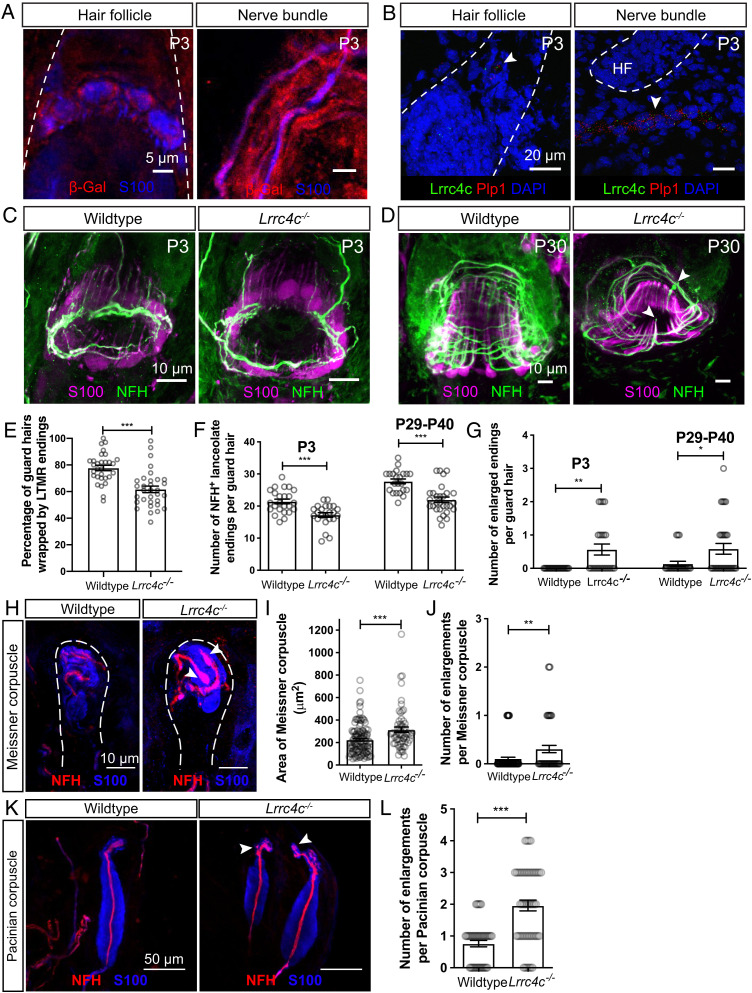
NGL-1 is expressed in terminal Schwann cells and contributes to the formation of lanceolate endings, Meissner corpuscles, and Pacinian corpuscles. (*A*) Wholemount IHC images of P3 back hairy skin sections (*n* = 3 animals), showing that β-Gal (red) is detected in S100^+^ terminal Schwann cells and nonmyelinating Schwann cells (blue). Hair follicles are outlined with white dotted lines. Skin from wild-type animals was used as negative controls and no β-Gal signal was observed. (*B*) Example images of smRNA-FISH for P3 back hairy skin sections (*n* = 3 animals). White arrowheads denote the detection of *Lrrc4c* mRNA (green) near *Plp1* mRNA (red). Hair follicles are outlined with white dotted lines. DAPI staining labels nuclei. (*C* and *D*) Wholemount immunostaining images of guard hairs in back hairy skin of P3 (*C*) and P30 (*D*) wild-type and *Lrrc4c^−/−^* animals. (*E*) Quantification of the percentage of guard hairs wrapped by NFH^+^ lanceolate endings in P0 wild-type (30 hair follicles from three animals) and *Lrrc4c^−/−^* animals (32 hair follicles from three animals). Each dot represents the result for one guard hair in back hairy skin. (*F* and *G*) Quantification of the number of NFH^+^ lanceolate endings (*F*) and the number of enlarged endings (*G*) in wild-type (24 hair follicles from four animals for P3; 22 hair follicles from three animals for P29–P40), and *Lrrc4c^−/−^* (23 hair follicles from three animals for P3; 29 hair follicles from four animals for P29–P40) animals. (*H*) Representative IHC images of forepaw glabrous skin sections of wild-type and *Lrrc4c^−/−^* animals. (*I* and *J*) Quantification of the area (*I*) and number of enlargements (*J*) of Meissner corpuscles in the wild-type (34 skin sections from four animals) and *Lrrc4c^−/−^* mice (32 skin sections from four animals). (*K* and *L*) Representative IHC images (*K*) and quantification of the number of enlargements per Pacinian corpuscle (*L*) of adult wild-type (20 corpuscles from three animals) and *Lrrc4c^−/−^* mice (36 corpuscles from three animals). Each dot represents a single Pacinian corpuscle. Student’s unpaired *t* test. *P < 0.05, **P < 0.01, ***P < 0.001.

Netrin-G ligand-1 (NGL-1), encoded by the gene *Lrrc4c*, is a type I transmembrane protein and a postsynaptic density (PSD)-95–interacting postsynaptic adhesion molecule that interacts with Netrin-G1 (30, 50). NGL-1 selectively binds to Netrin-G1 to activate downstream signaling that promotes synapse formation (32, 33). We therefore tested the hypothesis that NGL-1 functions as a ligand for Netrin-G1 in LTMR ending formation by determining the localization of NGL-1 and whether mice lacking NGL-1 exhibit the same LTMR deficits observed in *Ntng1* mutants. For these questions, we used *Lrrc4c* mutant mice in which the NGL-1 protein coding exon 3 of *Lrrc4c* was replaced with a β-geo cassette (50). This targeting strategy allowed us to examine the expression pattern of *Lrrc4c* in the skin by immunostaining for β-Gal. β-Gal signal was detected in S100^+^ TSCs and myelinating Schwann cells in hairy skin (Fig. 6*A*). In complementary experiments, single-molecule RNA fluorescent in situ hybridization (smRNA-FISH) was performed on P3 hairy skin using probes against *Lrrc4c* and the glial-specific gene *Plp1*. *Lrrc4c* signal was observed in close proximity to the *Plp1* signal around hair follicles and in the sensory nerve bundles near hair follicles (Fig. 6*B*). These findings indicate that *Lrrc4c* is expressed in TSCs and myelinating Schwann cells, while its receptor Netrin-G1 is expressed in LTMR axons, during the period of Netrin-G1–dependent end organ formation.

### *Lrrc4c* Regulates LTMR Ending Formation.

To determine whether *Lrrc4c* also regulates formation of lanceolate complexes, Meissner corpuscles, and Pacinian corpuscles, we examined hairy and glabrous skin of control and *Lrrc4c^−/−^* mice at different ages (Fig. 6 *C*–*J*). Similar to *Ntng1^−/−^* mice, *Lrrc4c^−/−^* mice showed a delay in the wrapping of lanceolate endings around guard hairs at P0 (Fig. 6*E*). In both P3 pups and young adults (P29–P40), lanceolate complexes around guard hairs in *Lrrc4c^−/−^* mice had fewer lanceolate endings, compared to those of littermate controls (Fig. 6 *C*, *D*, and *F*). The number of enlarged lanceolate endings was also increased in *Lrrc4c^−/−^* mice (Fig. 6*G*). We note that the lanceolate ending phenotype is milder than that observed in *Ntng1^−/−^* mice, suggesting that additional ligands might signal through Netrin-G1 to promote lanceolate ending formation. Furthermore, sparse labeling of individual Aβ RA-LTMR and Aδ-LTMR neurons in P3 hairy skin revealed that *Lrrc4c^−/−^* mice phenocopied the hair follicle innervation delay observed in *Ntng1^−/−^* mice (*SI Appendix*, Fig. S3 *E–H*). In addition, *Lrrc4c^−/−^* mice exhibited an increase in the area and number of enlargements of Meissner corpuscles (Fig. 6 *H–J*) as well as an increase in the number of enlargements in Pacinian corpuscles (Fig. 6 *K* and *L*), although these phenotypes were also milder that those observed in *Ntng1^−/−^* mice. These findings together with the *Lrrc4c* expression pattern suggest that NGL-1 in TSCs signals through Netrin-G1 in LTMR axons to promote formation of lanceolate ending complexes as well as Meissner corpuscles and Pacinian corpuscles.

## Discussion

Hair follicle lanceolate complexes are mechanically sensitive end organs that transform hair deflection into LTMR electrical signals. Lanceolate complexes are formed by Aβ RA-LTMR, Aδ-LTMR, and C-LTMR axonal endings and their associated TSCs. How this unique complex forms during development and the molecular players involved have been unclear. In this study, we combined genetic labeling of LTMRs and TSCs, histological analyses, and genetic manipulations to characterize developmental steps leading to the formation of Aβ RA-LTMR and Aδ-LTMR lanceolate ending complexes. We found that Aβ RA-LTMRs innervate hair follicles earlier than Aδ-LTMRs, and that branches of both LTMR subtypes exhibit robust pruning. The difference between these two LTMR subtypes in the timing of innervation was unexpected. Since the hair follicles innervated by Aβ RA-LTMRs (22% of Aβ RA-LTMR endings associate with guard hairs and 78% with awl/auchene hairs) are on average generated earlier, later than the hair follicles innervated by Aδ-LTMRs (23% of Aδ-LTMR endings associate with awl/auchene hairs and 77% with zigzag hairs) (6), the birthdate or maturation of their target hair follicle types could potentially contribute to the differences in innervation timing. Nascent lanceolate endings are associated closely with TSCs during lanceolate complex formation. Moreover, Netrin-G1 in sensory neurons acts to promote the formation of lanceolate complexes around hair follicles as well as Meissner corpuscles and Pacinian corpuscles innervated by Aβ RA-LTMRs. *Lrrc4c*, encoding NGL-1, a ligand for Netrin-G1, is expressed in TSCs and myelinating Schwann cells, and loss of *Lrrc4c* led to similar albeit less dramatic deficits in LTMR endings, revealing a critical role for neuron–glial interactions and NGL-1–Netrin-G1 signaling in LTMR end organ formation.

### Pruning of Touch Sensory Axons during Postnatal Development.

Across the nervous system, functional circuits are established through a progressive series of developmental processes, often including axonal branch elaboration and subsequently refined by regressive processes such as the pruning of excess axonal branches (51). Developmental neurite and synaptic pruning have been observed in many neuronal subtypes (52, 53); this ensures that unnecessary neurites or connections are removed to refine neural circuits for proper function. Using genetic labeling of developing Aβ RA-LTMRs and Aδ-LTMRs, we observed exuberant branching followed by pruning of mechanosensory neuron endings at two stages. First, excess axonal branches that do not innervate hair follicles are eliminated, which may contribute to the formation of precise functional receptive fields of LTMRs. Second, overproduced lanceolate endings that extend toward the basal side of the skin are pruned, which may ensure high sensitivity and, in the case of Aδ-LTMRs, directional selectivity (25), to hair deflection (3). The common pruning strategies across these two LTMR types suggest a common mechanism instructing this regressive event. If this is the case, then C-LTMRs, which also form lanceolate endings in hairy skin, may exhibit similar types of pruning during early postnatal development, and future experiments targeting developing C-LTMRs may help address this question. While TSCs are closely associated with sensory axons in the skin, it is unclear whether TSC–axon interactions contribute to axonal pruning. Another intriguing question is whether sensory neuron activity plays a role in instructing pruning. The axonal pruning observed here takes place around and shortly after birth, which roughly coincides with the period of time when evoked sensory activity in the mechanosensory system begins (22). It will be interesting to determine whether mechanosensory neuron activity contributes to pruning of LTMR endings in hairy skin.

### The Role of Netrin-G1 Signaling in Lanceolate Complex Formation.

The close association between developing TSCs and LTMRs suggests that TSCs may be instrumental in promoting LTMR axonal branching and lanceolate complex formation. In addition to LTMRs that form lanceolate endings, hair follicles are associated with additional LTMR ending types, notably Merkel cell innervating Aβ SA1-LTMRs and Aβ field-LTMRs that form circumferential endings. Future work should identify molecular players that direct formation of these axonal ending structures and how they contribute to the unique response properties of LTMRs to mechanical stimuli. Additionally, it is unclear whether loss of Netrin-G1 leads to any pruning deficits and whether changes in the pruning process could lead to a failure to innervate hair follicles. Live imaging of LTMR axon dynamics in the control and Netrin-G1 mutants would help to determine the cause of the observed hair follicle innervation failure.

Netrin-G1 receptors and at least one Netrin-G1 ligand, TSC-derived NGL-1, act to promote lanceolate ending formation. Interestingly, a recent finding shows that microglia accumulation around subcerebral projection axons depends on NGL-1–Netrin-G1 signaling (29), suggesting a general role for Netrin-G1 in mediating neuron–glial cell communication. We speculate that, in addition to NGL-1, other ligands signal through Netrin-G1, as *Lrrc4c* mutants exhibited a weaker phenotype than the *Ntng1* mutants. How Netrin-G1 signaling could mediate extension and structural integrity of lanceolate endings remains to be determined. One possibility is that NGL-1–Netrin-G1–mediated adhesion between axon terminals and TSCs may physically stabilize nascent lanceolate endings as they branch from basal circumferential axons. Another possibility is that Netrin-G1 signaling within axons promotes branching around the follicle. However, because Netrin-G1 is a GPI-anchored protein without an intracellular domain (52), it may function together with a coreceptor to activate downstream signaling within lanceolate processes. Thus, identification of Netrin-G1 coreceptors and understanding Netrin-G1–mediated intracellular signaling mechanisms may help to explain how Netrin-G1 promotes lanceolate ending formation. Any putative Netrin-G1 coreceptor is likely expressed in LTMRs and thus present in our LTMR sequencing datasets (45, 46). Nevertheless, the discovery of a function for Netrin-G1 signaling in lanceolate endings, Meissner corpuscles, and Pacinian corpuscles establishes a critical role for neuron–glia interactions and an intercellular molecular dialogue instructing the formation of cutaneous mechanosensory end organs.

## Materials and Methods

For a complete description of materials and methods, please refer to *SI Appendix*.

All procedures were conducted according to animal protocols approved by the Harvard Medical School Institutional Animal Care and Use Committee.

### Wholemount Immunostaining.

The back hairy skin samples were washed every 30 to 60 min with 0.3% Triton X-100 in phosphate-buffered saline (PBS) (0.3% PBST) for 5 to 8 h, incubated with primary antibodies in 0.3% PBST with 5% serum and 20% dimethyl sulfoxide (DMSO) for 3 to 5 d at room temperature. Skins were then washed every 30 min with 0.3% PBST for 5 to 8 h, incubated with secondary antibodies in 0.3% PBST with 5% serum and 20% DMSO for 2 to 3 d, and washed every 30 to 60 min with 0.3% PBST for 5 to 8 h. The samples were dehydrated in 50%, 75%, and 100% methanol for 10 min each before transferring them to 100% methanol. The skin was cleared with BABB (1 part benzyl alcohol:2 parts benzyl benzoate) for 5 min before it was mounted for confocal imaging. Guard hairs were identified by the presence of Merkel cells (TROMA-I positive).

## Supplementary Material

Supplementary File

## Data Availability

All study data are included in the article and/or supporting information.
